# Research trends and hotspots of liposomal bupivacaine: a bibliometric analysis from 2000 to 2025

**DOI:** 10.3389/fmed.2026.1955952

**Published:** 2026-10-13

**Authors:** Yining Wang, Ting Chen, Ying Zhang, Jingchao Kang, Mingdong Zhao, Duoqin Huang, Jinguo Yang

**Affiliations:** 1 Department of Anesthesiology, Sinopharm Dongfeng General Hospital Affiliated to Hubei University of Medicine, Shiyan, Hubei, China; 2 Department of Breast and Thyroid Surgery, Sinopharm Dongfeng General Hospital Affiliated to Hubei University of Medicine, Shiyan, Hubei, China; 3 The First Clinical Medical College, Gannan Medical University, Ganzhou, Jiangxi, China

**Keywords:** bibliometric analysis, drug delivery, liposomal bupivacaine, postoperative pain, regional anesthesia

## Abstract

**Background:**

Postoperative pain remains a persistent challenge in perioperative care, frequently contributing to delayed patient mobilization, hindered functional recovery, and increased opioid consumption. Liposomal bupivacaine was developed to extend the release duration of local anesthetics following a single-dose administration; however, its clinical utility exhibits considerable variability across surgical procedures, block techniques, and comparator regimens. From a bibliometric perspective, this study aims to systematically investigate the overall developmental trajectory, intellectual structure, and emerging research frontiers of liposomal bupivacaine.

**Methods:**

A comprehensive literature search was performed in the Web of Science and Scopus databases for studies on liposomal bupivacaine published between 2000 and 2025. After deduplicating and merging records from both databases using a custom script, the consolidated dataset was analyzed using VOSviewer, CiteSpace, and the Bibliometrix R package.

**Results:**

A total of 3,552 publications were included in this study. The annual publication volume increased markedly between 2020 and 2025, reaching its peak in 2025. In terms of geographical and institutional distribution, the United States dominated the field, ranking first with 2,075 publications and 63,218 total citations; China ranked second with 565 publications, though its international co-authorship rate was only 13.23%. Major contributions were made by leading institutions such as the Mayo Clinic. At the author level, De Paula demonstrated the highest publication output and network betweenness centrality, Urman recorded the highest average citations per paper, and Onel accumulated the highest total citations; collectively, these three scholars constituted the intellectual core of the field with the most prominent contributions. Regarding journal distribution, The Journal of Arthroplasty ranked first in productivity, whereas Advanced Drug Delivery Reviews garnered the highest cumulative citations, reflecting distinct emphases on clinical translation and basic pharmaceutical dissemination, respectively. Co-citation clustering identified five major thematic domains: arthroplasty, liposomal bupivacaine, fascial plane blocks, breast reconstruction, and local anesthetic delivery. “Extended-release bupivacaine” emerged as a core keyword characterized by high occurrence and high centrality. Concurrently, “erector spinae plane block” and “autologous breast reconstruction” have surged rapidly in recent years, demonstrating an ongoing thematic shift toward procedure-specific applications.

**Conclusion:**

This study represents the first quantitative bibliometric analysis of liposomal bupivacaine, revealing an evolutionary shift in research focus from early investigations centered on pharmacokinetics and formulation to diversified, procedure-specific clinical applications. Future studies should prioritize expanding international collaborative networks and further exploring emerging techniques, such as fascial plane blocks, across understudied surgical populations. This research roadmap provides directional guidance for investigators, clinicians, and funding agencies alike.

## 1 Introduction

Postoperative pain is still a common challenge in perioperative care. It can delay mobilization, disturb sleep, reduce patient satisfaction, and increase the need for rescue opioids. Recent guidelines and consensus statements therefore recommend planned multimodal analgesia rather than a simple rescue-opioid approach (1, 2). This change has also renewed interest in regional anesthesia and local anesthetic techniques. These methods can reduce nociceptive input near the surgical site or peripheral nerves and may fit well within opioid-sparing recovery pathways (3). Their value is especially clear when analgesia has to support early rehabilitation rather than only reduce pain scores at rest.

Liposomal bupivacaine is one such strategy. It is an extended-release local anesthetic formulation designed for postsurgical analgesia (4). Its clinical rationale is straightforward: multivesicular liposomes release bupivacaine gradually, aiming to extend analgesia after a single administration (4). This feature may be useful when continuous catheters are impractical or when early postoperative pain is expected to last beyond the usual duration of plain bupivacaine. The formulation also links two research traditions that are often discussed separately: sustained drug delivery and bedside regional analgesia. Even so, prolonged drug release does not guarantee better analgesia in every setting. Volunteer and phase I studies have shown that the pharmacodynamic profile of liposomal bupivacaine can be variable, especially when it is used alone for peripheral nerve blockade (5). Clinical evidence has also remained procedure-specific. Recent meta-analyses reported neutral or limited advantages in abdominal surgery and some orthopedic perineural applications (6–8). Other syntheses suggested more favorable effects in selected shoulder, spine, and thoracic contexts (7, 9, 10). Randomized trials have added a similar degree of nuance. Intercostal nerve block in thoracoscopic surgery and erector spinae plane block in open spinal surgery showed improved short-term analgesic or opioid-sparing outcomes in some studies (11, 12). In lumbar fusion, however, comparisons with wound infiltration showed only small short-term advantages (13). Differences in surgical trauma, block location, comparator drugs, and outcome timing may partly explain why conclusions diverge. These findings indicate that liposomal bupivacaine has moved beyond a single formulation question. It now spans drug delivery, surgical infiltration, regional anesthesia, opioid-sparing analgesia, and patient-centered recovery.

Traditional reviews and meta-analyses are useful for evaluating efficacy, but they do not fully describe how a research field is organized. Bibliometric analysis can fill this gap by mapping publication growth, collaboration networks, influential references, journals, and keyword evolution (14, 15). Related bibliometric studies have already examined postcesarean analgesia, ropivacaine for postoperative pain, and opioid-free anesthesia (16–18). These studies show how pain-management research shifts over time toward nerve blocks, multimodal regimens, enhanced recovery, and opioid-sparing care. A similar map is needed for liposomal bupivacaine because its literature includes pharmacology, surgery, anesthesia, and health-outcome studies. In recent years, substantial progress has been made globally in the investigation of liposomal bupivacaine. Nonetheless, a comprehensive bibliometric evaluation remains lacking to systematically address the following specific questions: (1) How did publication output and citation activity change between 2000 and 2025? (2) Which countries, institutions, and authors contributed most strongly, and how were they connected? (3) Which references, journals, and thematic clusters formed the intellectual base of the field? (4) Which keywords and recent bursts indicate expanding research domains, and what evidence boundaries should be applied when interpreting those domains? To address these questions, this study employs VOSviewer, CiteSpace, and the Bibliometrix R package to conduct a quantitative bibliometric analysis, aiming to delineate the evolutionary trajectory, collaboration landscapes, and thematic shifts in this domain, thereby providing directional references and strategic guidance for future investigations.

## 2 Materials and methods

### 2.1 Data collection

To ensure the reliability of the research content, this study adopts a method that integrates the WoS and Scopus databases for bibliometric visualization analysis. Data retrieval was conducted on both WoS and Scopus databases on July 20, 2026. The search strategy for WoS was {[ALL=(Liposomal bupivacaine)] OR ALL=(Bupivacaine liposome) OR ALL=(Exparel)] AND [DT = (Article OR Review)}AND [Time span = (January 2000 to December 2025)]AND. To ensure consistency, the search strategy used for Scopus was [ALL(”Liposomal bupivacaine”) OR ALL(”Bupivacaine liposome”) OR ALL(”Exparel”)] AND [DOCTYPE(ar) OR DOCTYPE(re)] AND (PUBYEAR > 1999 AND PUBYEAR < 2026].

### 2.2 Inclusion and exclusion criteria

This study included only English-language publications for analysis. Through the above searches, a total of 1152 relevant articles were retrieved from the WoS database, including 913 research papers and 239 reviews. From the Scopus database, 3,406 relevant articles were retrieved, including 2,379 research papers and 1,027 reviews.

Records retrieved from both databases were harmonized to the WoSCC structure and deduplicated using DOI matching, followed by normalized title, first-author, and publication-year checks for records without a DOI. Candidate matches were reviewed against available bibliographic identifiers, and Scopus secondary affiliations were mapped to primary institutions; obvious spelling and abbreviation variants were consolidated. After 1,006 cross-database duplicates were removed, 3,552 records remained. The process of including and excluding relevant literature is shown in Figure 1.

**Figure 1:**
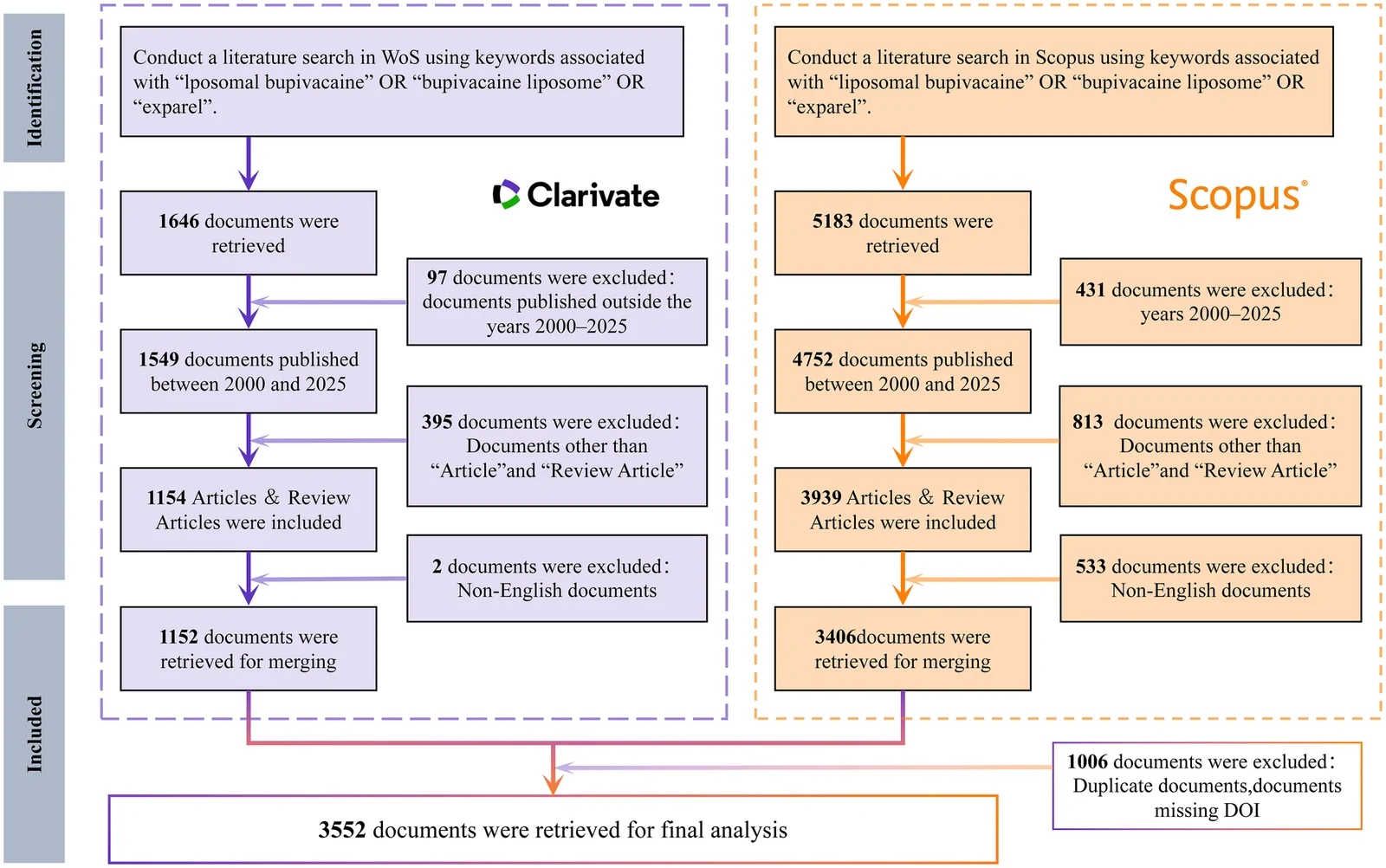
Flowchart of literature inclusion.

### 2.3 Data analysis and visualization

The merged dual databases were subjected to bibliometric analysis and visualization using VOSviewer (version 1.6.20), CiteSpace (version 6.4R1), Excel (version 2024), and the R package “Bibliometrix” (version 3.2.1).

VOSviewer was used to construct and visualize co-authorship networks for countries, institutions, and authors, as well as keyword co-occurrence networks. Publication counts and total link strength were used to describe contribution and collaboration structure, while keyword occurrence and centrality were used to describe thematic connections.

CiteSpace was used for co-cited reference clustering, citation-burst detection, and timeline visualization. Bibliometrix was used for corpus-level descriptive indicators, annual output, document types, and supplementary statistics. Descriptive counts and document types were cross-checked against the harmonized corpus, while network-specific outputs were interpreted within their own software definitions. The three tools were applied to the same harmonized 3,552-record corpus, but their outputs were treated as complementary because they use different algorithms, thresholds, and weighting conventions.

The Bibliometrix package, a bibliometric tool based on R, includes a wide range of effective statistical algorithms and high-quality examples. This tool was utilized for bibliometric statistics and supplementary analyses in the study.

## 3 Results

### 3.1 Bibliometric analysis of annual publications

Table 1 summarizes the general characteristics of the dataset. A total of 3,552 documents related to liposomal bupivacaine were retrieved from 947 sources during 2000–2025. The annual growth rate was 18.73%, the document average age was 5.56 years, and the average citation count per document was 30.57. The dataset contained 153,187 cited references, 12,364 Keywords Plus terms, and 5,262 author keywords. In terms of authorship, 17,414 authors contributed to the corpus, including 95 authors of single-authored documents. Collaboration was common, with 5.98 co-authors per document and an international co-authorship rate of 13.23%. Articles constituted the main document type, with 3,317 records, followed by 235 reviews.

**Table 1:** General information.

Description	Results
**MAIN INFORMATION ABOUT DATA**	
Timespan	2000:2025
Sources (Journals, Books, etc)	947
Documents	3,552
Annual Growth Rate %	18.73
Document Average Age	5.56
Average citations per doc	30.57
References	1,53,187
**DOCUMENT CONTENTS**	
Keywords Plus (ID)	12,364
Author's Keywords (DE)	5,262
**AUTHORS**	
Authors	17,414
Authors of single-authored docs	95
**AUTHORS COLLABORATION**	
Single-authored docs	111
Co-Authors per Doc	5.98
International co-authorships %	13.23
**DOCUMENT TYPES**	
article	3,317
review	235

The annual publication curve in Figure 2 shows a long early stage of low output followed by a rapid expansion after 2012. From 2000 to 2011, yearly output remained below 20 documents, ranging from 5 publications in 2007 to 17 publications in 2010. Output increased to 41 documents in 2012 and then rose more consistently after 2014, reaching 82 publications in 2015, 119 in 2016, 178 in 2017, and 238 in 2018. The field entered a high-output phase after 2019, with 303 publications in 2019, 335 in 2020, 395 in 2021, 420 in 2022, 343 in 2023, 383 in 2024, and 512 in 2025. The highest annual output was therefore observed in 2025, indicating that research activity on liposomal bupivacaine remained active at the end of the study period. Average citations per year fluctuated more sharply than publication output. Early peaks appeared in 2005 and 2013, with average annual citation values of 16.90 and 20.18, respectively, whereas several later high-output years had lower average annual citation values. For example, the values were 4.26 in 2020, 3.52 in 2021, 2.79 in 2022, 2.94 in 2023, 2.36 in 2024, and 1.66 in 2025. This pattern indicates that the recent expansion of publications was driven mainly by growing research volume, while the citation accumulation of newer papers remained limited by their shorter citation window.

**Figure 2:**
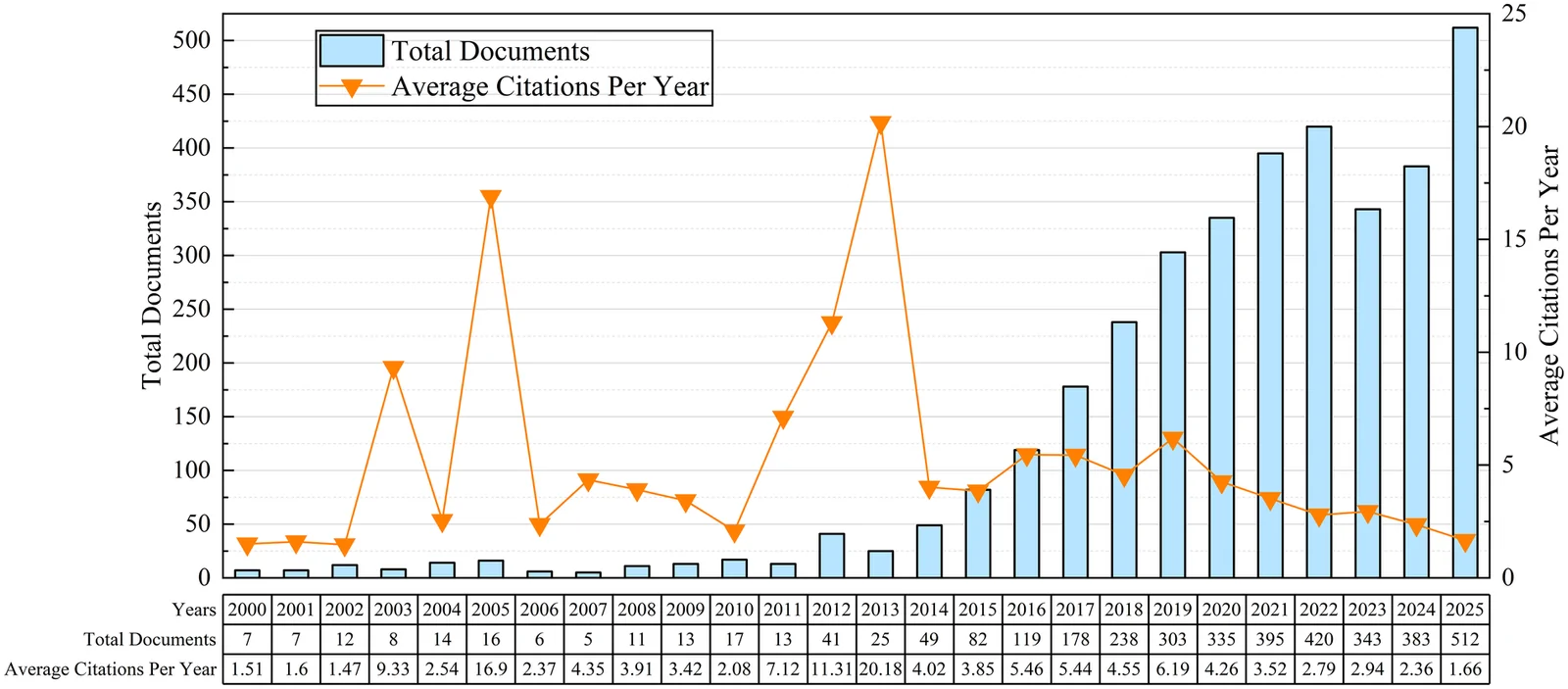
Annual publication output and citation trends in liposomal bupivacaine research. Annual document counts and average citations per year from 2000 to 2025 are presented on dual axes.

### 3.2 Bibliometric analysis of countries and institutions

Country-level output was concentrated in a United States-centered core but became more internationally distributed over time. The United States contributed 2,075 publications and had the highest total link strength (TLS=418), more than three times China's 565 publications; Canada and the United Kingdom followed with 178 and 127, respectively. Citation impact did not mirror publication volume: Canada and the United Kingdom averaged 95.71 and 74.76 citations per document, compared with 30.47 for the United States and 16.26 for China. Canada and the United Kingdom also showed relatively strong collaborative links (TLS=180 and 170), indicating that network roles were not explained by publication volume alone. These raw averages are sensitive to publication age and highly cited outliers, but they show that productivity and citation accumulation followed different national patterns. Keyword profiles further distinguished the portfolios: U.S. studies emphasized postoperative pain, regional anesthesia, opioids, knee arthroplasty, enhanced recovery, and multimodal analgesia, whereas Chinese studies more often combined arthroplasty and meta-analysis with intercostal, erector spinae, and transversus abdominis plane blocks, thoracoscopic surgery, and dexamethasone. Canada and the United Kingdom repeatedly featured enhanced recovery, arthroplasty, and regional or peripheral nerve blocks.

Figures 3A,B places the United States at the center of a network linking North America, Europe, Asia, and South America. The temporal overlay suggests later expansion toward Asian contributors, with mean publication years of 2020.34 for the United States, 2022.11 for China, 2021.75 for India, and 2021.15 for Turkey. Thus, international participation broadened beyond the original Western core while remaining organized around a dominant U.S. node; country-level prominence was shaped jointly by output, citations, collaboration, and thematic specialization.

**Figure 3:**
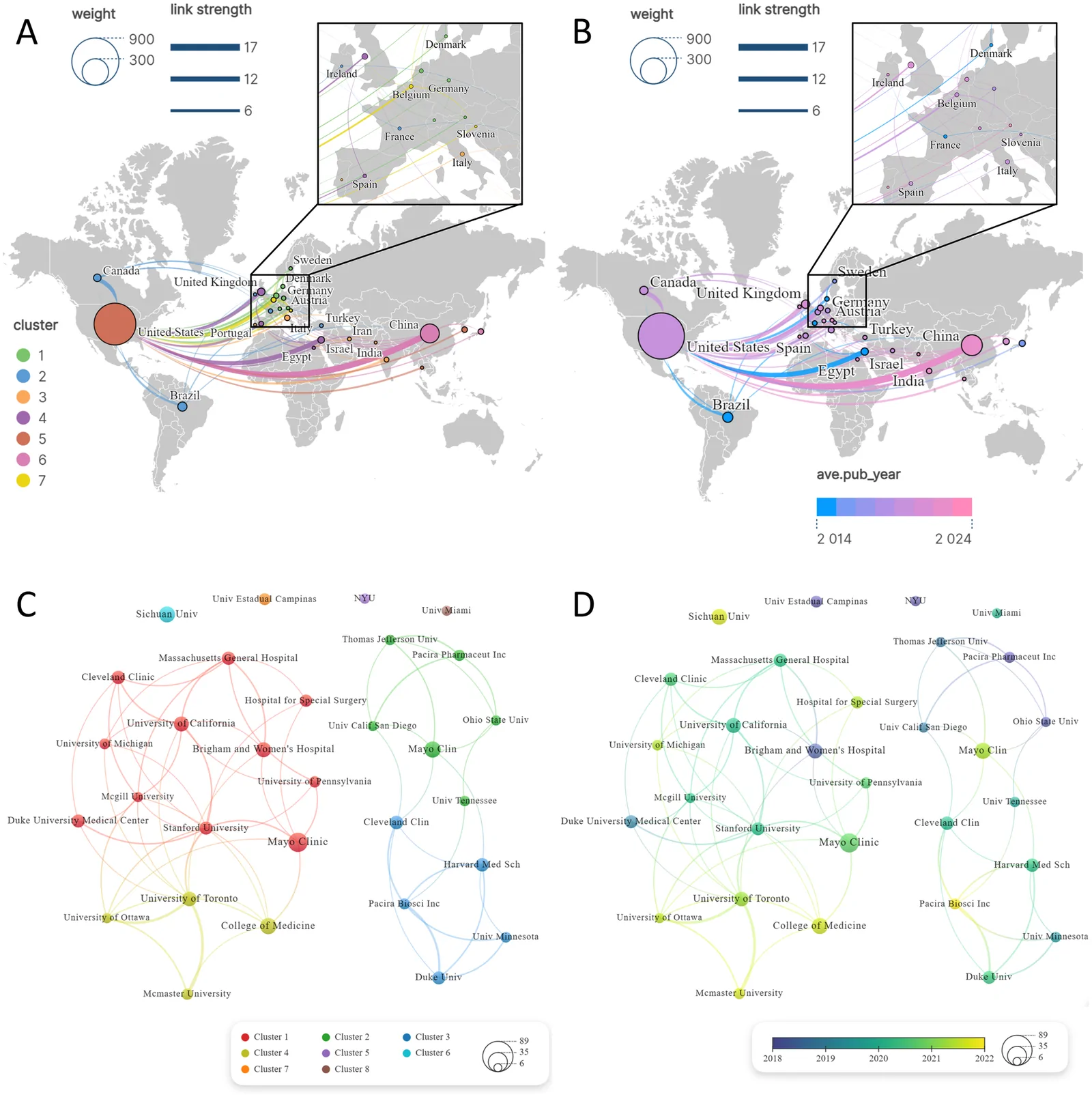
Geographic and institutional collaboration in liposomal bupivacaine research. **(A)** Country collaboration network, mapping research clusters and collaborative link strengths among the most productive countries. **(B)** Temporal overlay of country collaboration, showing average publication years and the recent expansion of Asian contributors. **(C)** Institutional collaboration network, identifying major clusters among universities, medical centers, and industry organizations. **(D)** Temporal overlay of institutional collaboration, displaying average publication years and the emergence of recently active institutions.

Institutional production showed a similar concentration in major medical centers and universities. Mayo Clinic led with 105 documents, followed by Cleveland Clinic (19), Duke University (20), and Sichuan University (21). Citation performance again diverged from output: the University of California and Massachusetts General Hospital averaged 154.86 and 136.24 citations per document, respectively, whereas Mayo Clinic averaged 19.96. The University of Toronto had the strongest institutional collaboration links (TLS=29), followed by Duke and Stanford (TLS=21 each). Affiliation-level keywords indicated complementary portfolios: Mayo, Cleveland, and Duke emphasized postoperative pain, multimodal analgesia, arthroplasty, regional anesthesia, and nerve blocks; California and Massachusetts General focused more on colectomy or proctectomy, ileus, and thoracic or cardiac surgery; Toronto highlighted shoulder and adductor-canal blocks and rebound pain; and Stanford linked transversus abdominis plane blocks with enhanced recovery. These contrasts suggest that institutional influence reflected different combinations of productivity, citation accumulation, collaboration, and clinical specialization.

The institutional maps in Figures 3C,D showed several partially connected clusters rather than one integrated network, including North American groups, a Mayo-Pacira cluster, and a Canadian group. Sichuan University and Pacira Biosciences appeared toward the recent end of the overlay, indicating continued expansion of the institutional landscape. The pattern nevertheless remains sensitive to affiliation normalization and network thresholds, so apparent institutional prominence should be interpreted as a feature of the mapped research structure rather than a standalone measure of scientific or clinical importance.

### 3.3 Bibliometric analysis of active authors

Author activity was concentrated among a small group, but productivity, citation accumulation, and collaboration represented different dimensions of influence. De Paula led publication output (40 documents, 1,092 citations, TLS=57), followed by Franz-Montan (20 documents, 489 citations, TLS=42). Kohane also published 20 documents but accumulated 1,162 citations (58.10 per document), while Urman had the highest average (131.58) and Onel the highest total citations (1,751) among the listed authors. Keyword profiles further separated research roles: De Paula and Franz-Montan were associated mainly with liposomes, local anesthetics, and drug delivery; Kohane with controlled release, biocompatibility, and toxicity; and later clinical authors with multimodal analgesia, regional blocks, arthroplasty, breast surgery, abdominal or chest-wall blocks, patient-reported outcomes, and enhanced recovery. The author portfolio therefore connected formulation research with procedure-specific perioperative applications. Figures 4A,B shows that collaboration was organized around a core centered on De Paula and Franz-Montan, linked to De Araujo and Saia Cereda. Their TLS values were 57, 42, and 29, respectively. Kohane had high productivity and citation totals but no displayed TLS, suggesting a more independent authorship pattern within the selected threshold. Other clusters, including Joshi, Kaye, Mariano, and Urman, were more closely aligned with clinical anesthesia and perioperative analgesia. The network thus combined a stable formulation-oriented core with more dispersed clinical groups.

**Figure 4:**
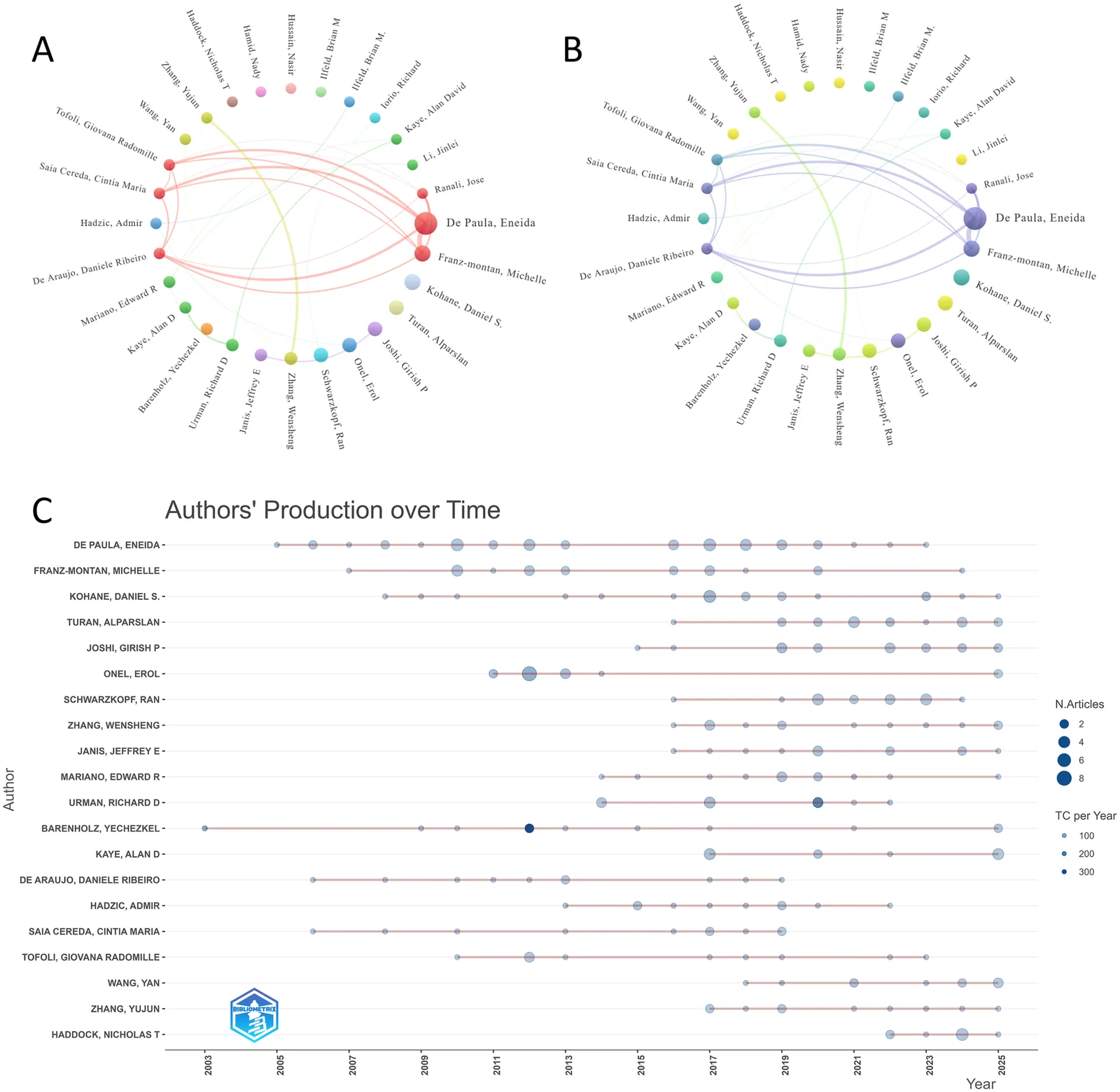
Author collaboration and publication dynamics in liposomal bupivacaine research. **(A)** Author collaboration network, showing research clusters and the prominent group centered on De Paula and Franz-Montan. **(B)** Temporal overlay of author collaboration, indicating average publication years and shifts from formulation-focused to clinically oriented contributors. **(C)** Authors’ production over time, showing annual output and citation intensity for leading authors from 2003 to 2025.

The production-over-time plot in Figure 4C showed both continuity and thematic expansion. De Paula and Franz-Montan maintained activity from the mid-2000s, with De Paula showing local output peaks in 2010 and 2017, whereas Barenholz represented earlier foundational work with a citation peak in 2012. Franz-Montan also showed recurring activity across 2010, 2012, 2017, and 2019. Authors such as Turan, Schwarzkopf, Wang, Zhang, and Haddock became more active after 2017. This temporal pattern suggests a gradual transition from formulation-oriented authorship toward a broader clinical perioperative research community.

### 3.4 Bibliometric analysis of published journals

Journal output was distributed across anesthesiology, pain medicine, surgery, orthopedics, and pharmaceutics. As shown in Table 3 and Figure 5A, Journal of Arthroplasty led publication volume (84 articles; 2,991 citations), followed by Regional Anesthesia and Pain Medicine (63; 2,565) and Anesthesia and Analgesia (56; 3,355). Other productive sources included Journal of Shoulder and Elbow Surgery (51 articles), Journal of Pain Research (22), Plastic and Reconstructive Surgery (23), Journal of Cardiothoracic and Vascular Anesthesia (24), Anesthesiology and Journal of Clinical Anesthesia (37 each), and International Journal of Pharmaceutics (25). Among the leading sources, Anesthesiology had the highest 2024 impact factor (9.1), followed by International Journal of Pharmaceutics (5.2) and Journal of Clinical Anesthesia (5.1). The publication pattern therefore linked procedure-oriented clinical research with formulation and delivery science.

**Table 3:** Top 10 most productive journals.

Journal	Citations	Articles	IF^2024^
Journal of Arthroplasty	2,991	84.00	3.8
Regional Anesthesia and Pain Medicine	2,565	63.00	3.5
Anesthesia and Analgesia	3,355	56.00	3.8
Journal of Shoulder and Elbow Surgery	1,204	51.00	2.9
Journal of Pain Research	1,289	50.00	2.5
Plastic and Reconstructive Surgery	1,303	45.00	3.4
Journal of Cardiothoracic and Vascular Anesthesia	741	40.00	2.1
Anesthesiology	2,360	37.00	9.1
Journal of Clinical Anesthesia	885	37.00	5.1
International Journal of Pharmaceutics	1,539	34.00	5.2

**Figure 5:**
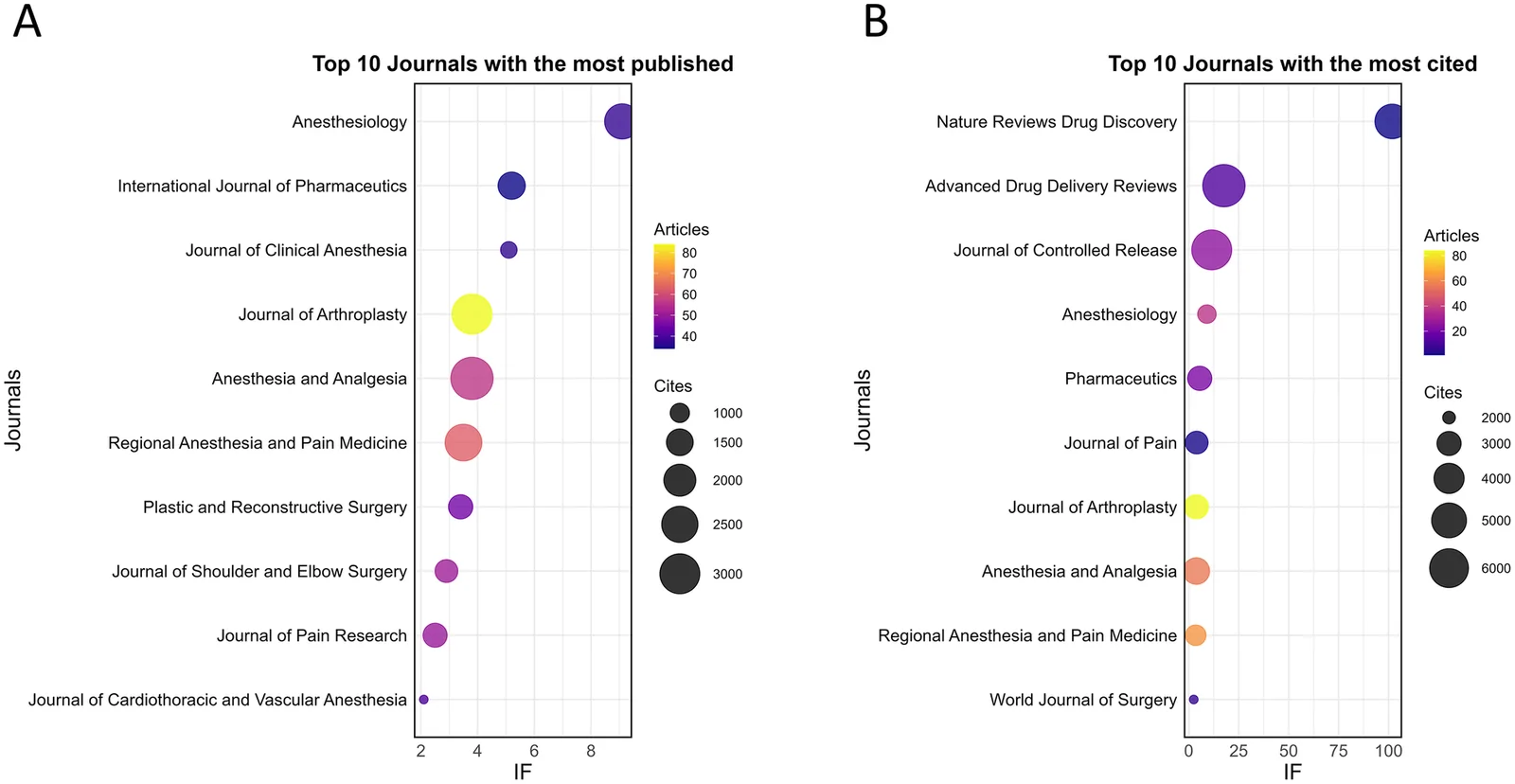
Productive and influential journals in liposomal bupivacaine research. **(A)** Top ten journals by publication output, with impact factor on the *x*-axis, citation count represented by bubble size, and article count by color. **(B)** Top ten journals by total citations, comparing impact factors, citation volume, and publication output across leading sources.

Citation rankings differed from publication rankings. Advanced Drug Delivery Reviews accumulated 6,998 citations from 12 articles, while Journal of Controlled Release received 6,295 citations from 25 articles. Nature Reviews Drug Discovery had only one article but 4,922 citations and the highest impact factor (101.8). Anesthesia and Analgesia, Pharmaceutics, Journal of Arthroplasty, Journal of Pain, Regional Anesthesia and Pain Medicine, Anesthesiology, and World Journal of Surgery also appeared among the ten most cited journals. This distribution indicates that citation influence was shared between high-volume perioperative venues and smaller drug-delivery or pharmacology literatures. Thus, publication volume and accumulated citation influence captured related but distinct aspects of journal prominence.

Figure 5 reinforces this two-part journal structure. In the publication-output plot, Journal of Arthroplasty, Regional Anesthesia and Pain Medicine, and Anesthesia and Analgesia were prominent because of article count; in the citation plot, Advanced Drug Delivery Reviews and Journal of Controlled Release stood out because of accumulated citations despite fewer papers. The field therefore occupied two connected publication ecosystems, with clinical outlets disseminating procedure-specific analgesia research and pharmacology-oriented outlets supplying the formulation and delivery framework.

### 3.5 Co-cited reference analysis

The CiteSpace co-citation network in Figure 6A contained 1,351 nodes and 2,827 edges, with a density of 0.0031. Its largest connected component included 573 nodes (42%), and clustering showed high modularity (Q = 0.8992), weighted mean silhouette (S = 0.9366), and harmonic mean (Q,S) = 0.9175. These values indicate a coherent but internally differentiated knowledge structure. Major clusters covered total knee and total joint arthroplasty, liposomal bupivacaine, plane blocks, autologous breast reconstruction, local-anesthetic delivery, rescue analgesia, and pegylated liposomes. The cluster structure consequently linked the formulation basis of the field with procedure-specific and recovery-oriented applications.

**Figure 6:**
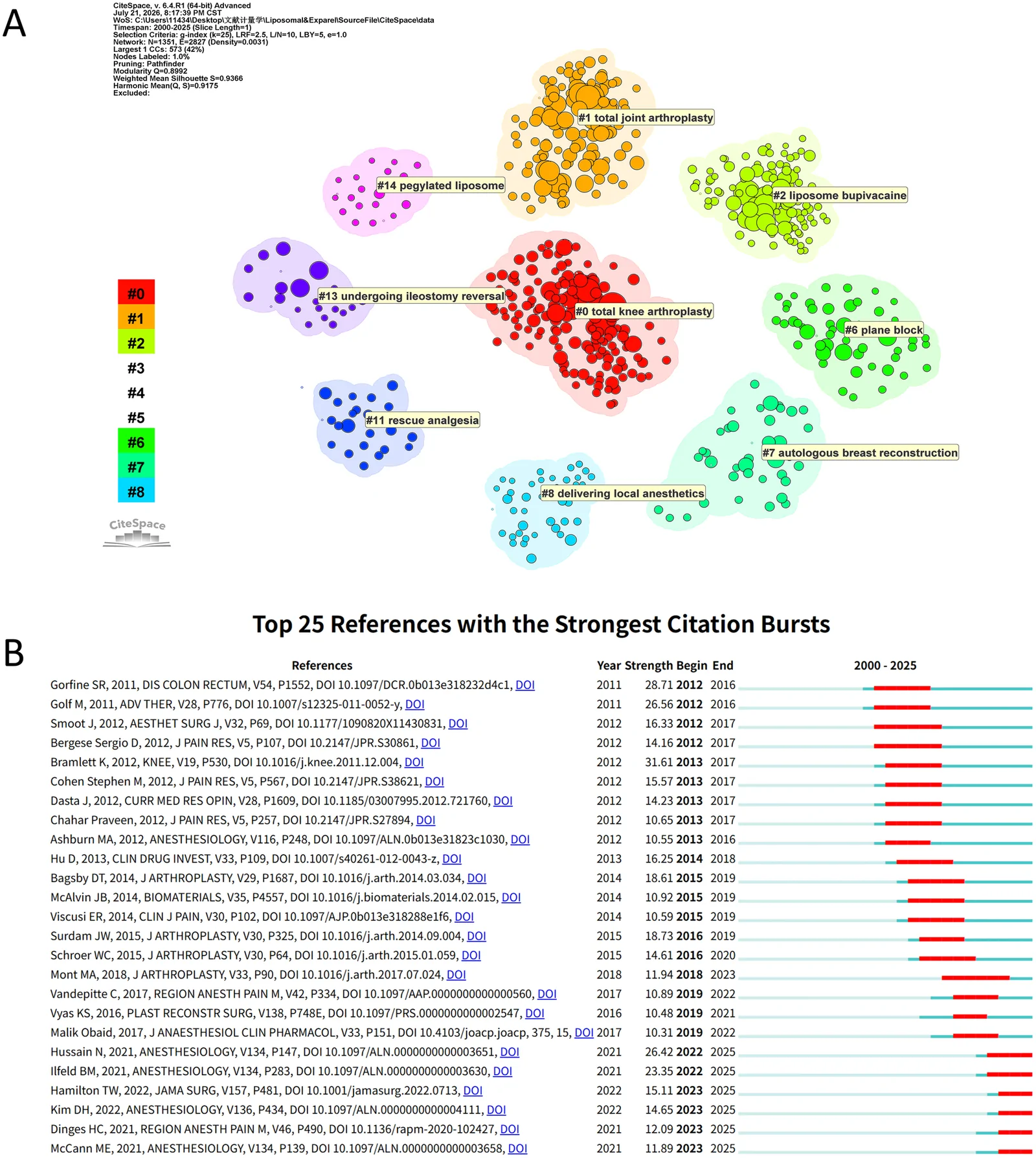
Co-cited reference structure and citation bursts in liposomal bupivacaine research. **(A)** Co-cited reference clustering network, revealing themes including arthroplasty, liposomal bupivacaine, plane blocks, breast reconstruction, and local anesthetic delivery. **(B)** Citation burst analysis of the top 25 references, showing periods of rapidly increasing scholarly attention between 2000 and 2025.

Table 2 lists the ten most frequently co-cited references in the CiteSpace network. Golf M (2011) ranked first (frequency=140), followed by Hu D (2013, 129), Hussain N (2021, 82), Schroer WC (2015, 81), Bagsby DT (2014, 77), Dasta J (2012, 74), Surdam JW (2015, 74), Grant GJ (2004, 70), Mont MA (2018, 69), and Vandepitte C (2017, 65). These frequencies indicate co-citation prominence within the mapped literature rather than ordinary citation counts or clinical effectiveness. The ranking combines an early phase 3 bunionectomy study, systematic evidence syntheses, arthroplasty trials, a colectomy comparison, and regional-block research. This mixture suggests that the field's shared intellectual base spans foundational efficacy and pharmacology evidence as well as procedure-specific comparative analgesia.

**Table 2:** Top 10 co-cited references by co-citation frequency.

Author	Citation information	Co-citation frequency
Golf M	2011,Adv Ther,Safety and efficacy of DepoFoam bupivacaine in bunionectomy: results of a randomized, double-blind, placebo-controlled, phase 3 study	140
Hu D	2013,Clin Drug Invest,Efficacy and safety of liposomal bupivacaine for postsurgical analgesia: a systematic review and meta-analysis	129
Hussain N	2021,Anesthesiology,Perineural Liposomal Bupivacaine Is Not Superior to Nonliposomal Bupivacaine for Postoperative Analgesia: A Systematic Review and Meta-analysis	82
Schroer WC	2015,J Arthroplasty,Does Liposomal Bupivacaine Reduce Pain and Inpatient Opioid Consumption in Total Knee Arthroplasty? A Prospective, Randomized, Double-Blind, Controlled Trial	81
Bagsby DT	2014,J Arthroplasty,Liposomal Bupivacaine Versus Traditional Periarticular Injection for Pain Control After Total Knee Arthroplasty	77
Dasta J	2012,Curr Med Res Opin,Bupivacaine liposome injectable suspension compared with bupivacaine HCl for the reduction of postsurgical pain in open colectomy: results from a multicenter, randomized, double-blind, paracetamol-controlled study	74
Surdam JW	2015,J Arthroplasty,The Use of Exparel (Liposomal Bupivacaine) to Manage Postoperative Pain in Unilateral Total Knee Arthroplasty Patients	74
Grant GJ	2004,Anesthesiology,Analgesic Efficacy of Liposomal Bupivacaine in a Rodent Model of Postoperative Pain	70
Mont MA	2018,J Arthroplasty,Local Infiltration Analgesia With Liposomal Bupivacaine Improves Pain Scores and Reduces Opioid Consumption After Total Knee Arthroplasty: Results of a Large Multicenter Study	69
Vandepitte C	2017,Region Anesth Pain M,Failure of Liposomal Bupivacaine to Extend Interscalene Brachial Plexus Blockade: A Prospective, Randomized, Double-Blind, Controlled Trial	65

Citation bursts in Figure 6B further showed a temporal change in the knowledge base. Early bursts, concentrated between 2012 and 2017, included Gorfine, Golf, Smoot, Bergese, Bramlett, Cohen, Dasta, Chahar, and Hu, reflecting attention to efficacy reviews, local infiltration, and foundational delivery studies. Later bursts extending into 2025 included clinical syntheses and active-comparator work by Hussain (strength=26.42), Ilfeld (23.35), Hamilton (15.11), Kim (14.65), Dinges (12.09), and McCann (11.89). The sequence suggests a shift from establishing prolonged delivery and early applications toward evaluating comparative strategies within procedure-specific perioperative care. Burst intensity identifies changing scholarly attention, rather than a hierarchy of clinical benefit.

### 3.6 Keyword analysis

The keyword network in Figure 7A and Table 4 contained 411 nodes and 1,091 edges (density=0.0129); its largest connected component included 324 nodes, or 78% of the network. “Extended release bupivacaine” was the most frequent and central term (341 occurrences; centrality=0.17), followed by “postoperative pain” (266; 0.14), “control postoperative pain” (236; 0.07), “knee arthroplasty” (195; 0.05), “regional anaesthesia” (192; 0.07), “plane block” (134; 0.08), “multimodal drug injection” (121; 0.13), and “enhanced recovery after surgery” (117; 0.04). Together, these terms connect sustained-release formulation with postoperative pain management, regional anesthesia, and procedure-specific recovery research rather than indicating a single dominant clinical application.

**Figure 7:**
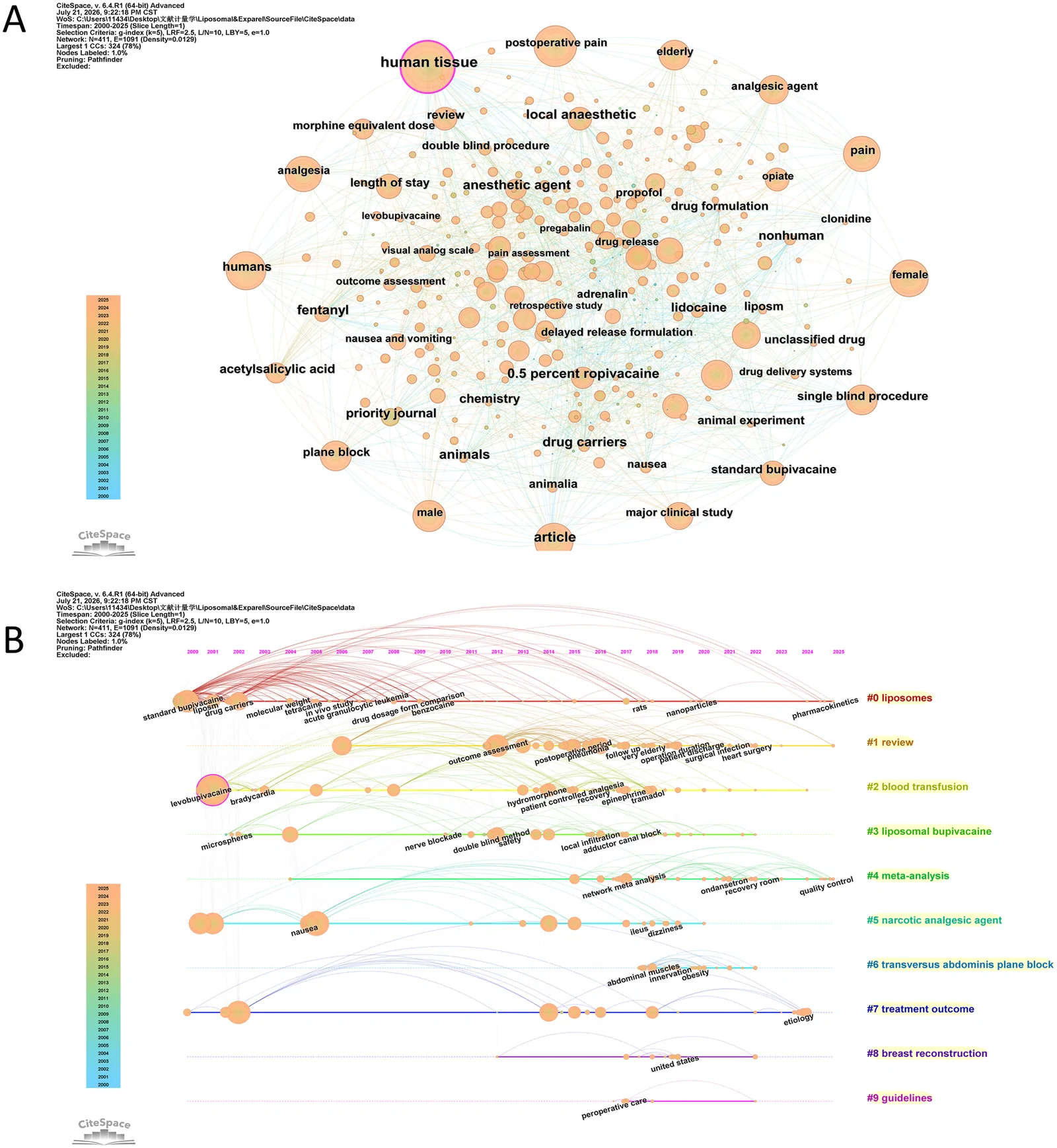
Keyword structure and thematic evolution in liposomal bupivacaine research. **(A)** Keyword co-occurrence network, showing frequency, centrality, and connections among formulation, postoperative pain, regional anesthesia, and drug-delivery terms. **(B)** Keyword timeline map, displaying thematic clusters and their evolution from formulation science toward regional blocks and perioperative outcomes.

**Table 4:** Top 25 most frequent keywords.

Keywords	Occurrences	Centrality	Year
extended release bupivacaine	341	0.17	2011
postoperative pain	266	0.14	2008
control postoperative pain	236	0.07	2015
knee arthroplasty	195	0.05	2012
regional anaesthesia	192	0.07	2003
plane block	134	0.08	2002
multimodal drug injection	121	0.13	2013
enhanced recovery after surgery	117	0.04	2016
postsurgical analgesia	83	0.06	2000
local anesthetics	79	0.16	2000
pain control	78	0.09	2014
drug delivery	76	0.13	2001
abdominis plane block	72	0.05	2016
local anesthetic	65	0.13	2000
erector spinae block	56	0.05	2019
length of stay	55	0.04	2012
total hip	55	0.03	2017
local anesthesia	54	0.05	2009
acute pain	54	0.06	2014
peripheral nerve block	49	0.05	2005
autologous breast reconstruction	48	0.01	2018
enhanced recovery	45	0.03	2017
adductor canal block	44	0.02	2015
tap block	42	0.02	2017
opioid consumption	41	0.05	2017

Centrality measures identified the terms that bridged formulation and clinical topics: “local anesthetics” (0.16), “postoperative pain” (0.14), and “drug delivery,” “local anesthetic,” and “multimodal drug injection” (0.13 each). Burst analysis showed early attention to “local anesthetics” (6.61, 2000–2011) and “drug delivery” (6.25, 2001–2013), followed by bursts for “liposome bupivacaine” (8.11, 2012–2016), “knee arthroplasty” (17.29, 2014–2018), “pain control” (8.93, 2016–2018), and “periarticular injection” (7.11, 2016–2019). More recent bursts for “erector spinae block” (4.96), “autologous breast reconstruction” (4.34), and “level iii” (4.03) in 2023–2025 suggest a shift toward newer regional techniques and procedure-specific outcome studies.

The timeline map in Figure 7B supports the same progression. Early clusters concerned liposomes, drug carriers, molecular properties, standard bupivacaine, tetracaine, microspheres, and levobupivacaine, reflecting formulation and pharmacologic foundations. Later clusters included treatment outcome, transversus abdominis plane block, breast reconstruction, guidelines, recovery, patient-controlled analgesia, local infiltration, adductor canal and other nerve blocks, meta-analysis, and perioperative care. The field therefore retained formulation science as a foundation while increasingly connecting it to regional techniques, evidence synthesis, and surgical recovery pathways.

## 4 Discussion

### 4.1 General information

This bibliometric analysis included 3,552 articles and reviews from 947 sources between 2000 and 2025. The annual growth rate of 18.73%, 153,187 cited references, and more than 17,000 contributing authors indicate that the indexed literature has broadened from formulation and drug-delivery work to a wider perioperative research field. The corresponding keyword profiles in Supplementary Tables S4–S6 connect this expansion with postoperative pain, regional anaesthesia, multimodal drug injection, enhanced recovery, and procedure-specific outcomes. This trajectory is consistent with guidance that places local and regional analgesia within multimodal, opioid-sparing pathways rather than treating any single agent as a complete solution (1–3), and with related bibliometric studies of regional techniques, long-acting local anesthetics, and opioid-free anesthesia (16–18).

Publication history suggests three overlapping phases. Output remained low from 2000 to 2011, when studies focused on carriers, release behavior, tissue exposure, and early infiltration. It rose after 2012 and accelerated after 2016 as liposomal bupivacaine entered arthroplasty, shoulder, abdominal, and enhanced-recovery studies. The peak of 512 papers in 2025 marks a high-output phase. Pharmacokinetic work had established prolonged absorption, while early reviews broadened proposed uses (4, 26). Recent studies increasingly compare prolonged exposure with plain bupivacaine, dexamethasone-containing mixtures, periarticular cocktails, or continuous catheters using pain, opioid, functional, and discharge-related outcomes. The lower citation averages of the newest papers are more consistent with citation latency than with reduced relevance.

Research production was geographically concentrated: the United States contributed 2,075 documents versus 565 from China and occupied the central collaboration position. Canada and the United Kingdom had higher average citations per document than the United States and China, but these raw averages remain sensitive to publication age and highly cited papers. The country, institutional, and author rankings and keyword profiles (Supplementary Tables S1–S6) identify the clinical questions attached to these portfolios: U.S. work clustered around postoperative pain, regional anaesthesia, opioids, knee arthroplasty, enhanced recovery, nerve blocks, and length of stay; Chinese work combined meta-analysis and arthroplasty with intercostal, erector spinae, and transversus abdominis plane blocks; Canadian and UK profiles emphasized enhanced recovery, arthroplasty, and regional or peripheral nerve blocks. Read alongside the comparative literature, this pattern places arthroplasty in a relatively mature but comparator-sensitive evidence domain, with randomized trials and meta-analytic syntheses yielding variable conclusions (27–38). At the institutional level, Mayo Clinic, Cleveland Clinic, and Duke University led production, whereas the University of California and Massachusetts General Hospital combined smaller outputs with higher average citations per document. The international co-authorship rate of 13.23% also supports broader pathway and cost comparisons across health systems.

The author and journal maps show a related translational division. De Paula, Franz-Montan, and Kohane were associated with liposome, local-anesthetic delivery, biomaterials, controlled release, and toxicity terms, whereas more recent clinical authors were associated with multimodal analgesia, opioid-sparing care, abdominal or chest-wall blocks, arthroplasty, breast surgery, patient-reported outcomes, and enhanced recovery (Supplementary Tables S3, S6). Formulation journals and clinical anesthesia, arthroplasty, shoulder, and plastic-surgery journals therefore form complementary parts of the field. The recent prominence of shoulder surgery, spine and erector spinae blocks, thoracic surgery, and breast reconstruction suggests expanding application domains; compared with arthroplasty, these areas remain less consolidated and more dependent on technique, dose, volume, background analgesia, and endpoint selection.

Co-citation structure reinforces this evidence gradient. Total knee and total joint arthroplasty formed the largest clinical clusters, while plane block, autologous breast reconstruction, and local-anesthetic delivery marked later diversification. Early bursts centered on efficacy reviews and periarticular infiltration; later bursts involved systematic reviews, active-comparator trials, and critical appraisals. The latter literature repeatedly found no consistent perineural advantage over nonliposomal local anesthetics (27, 28), and dexamethasone-containing comparators and trial-sequential analyses further narrowed the settings in which an advantage could be claimed (29). Thus, the most visible clinical hotspot is also a key area for resolving comparator and pathway effects.

Keyword results refine the framework. “Extended release bupivacaine” remained the central formulation term, while “postoperative pain,” “regional anaesthesia,” “knee arthroplasty,” “multimodal drug injection,” “enhanced recovery after surgery,” “length of stay,” and “opioid consumption” connected the formulation to procedure-specific recovery questions. The strongest burst for knee arthroplasty corresponds to the mature but inconsistent domain; recent bursts for erector spinae block and autologous breast reconstruction identify emerging domains whose evidence is less consolidated. Future studies should prioritize direct comparisons of liposomal bupivacaine with optimized plain bupivacaine, bupivacaine plus dexamethasone, and continuous catheters within standardized pathways. They should also prespecify block location, dose, volume, admixture, movement-evoked pain, opioid-related adverse effects, functional milestones, length of stay, readmission, and cost. For emerging spine, thoracic, and breast settings, multicenter designs would help determine whether apparent analgesic differences persist across operative techniques and recovery protocols.

### 4.2 Hotspot directions

The three directions identified by the hotspot analysis require different evidence standards. Formulation studies address pharmacokinetics and pharmacodynamics (30), with dose, admixture, systemic absorption, and block location shaping safety interpretation (31, 39). Procedure-specific studies test comparative effectiveness within optimized multimodal pathways (40, 41), while pooled and noninferiority analyses define the uncertainty that remains (32, 33). Block-focused studies examine anatomical and technical moderators, including recovery endpoints (34, 35), with pooled opioid findings remaining nonuniform (36, 37). The following subsections therefore focus on points of disagreement and on design features needed to distinguish a pharmacologic signal from a procedure-level recovery benefit.

#### 4.2.1 Extended-release formulation and local-anesthetic delivery

Formulation studies provide the mechanistic basis for prolonged exposure (25, 38). The multivesicular carrier can release bupivacaine over time rather than as a rapid bolus (26, 30), but release depends on injection site, tissue perfusion, dose, dilution, and carrier disruption. Direct volunteer and phase I studies should therefore link plasma pharmacokinetics to sensory or motor effects rather than infer block duration from drug presence alone. Safety requires parallel assessment of total dose, admixture, vascularity, and local tissue conditions (42, 43); slow release does not eliminate systemic toxicity risk (31, 44). Phase III sciatic-block research illustrates the need for indication-specific dosing (39). The unresolved clinical comparison is whether this pharmacologic profile adds benefit over optimized plain bupivacaine, dexamethasone-containing regimens, or catheter-based delivery. Existing syntheses suggest small or inconsistent perineural differences (27, 28), with a narrower apparent gap when dexamethasone is used (29, 45). Future studies should prespecify PK/PD endpoints and report particle integrity, free-drug and nerve exposure, admixture, and patient factors that may alter release.

#### 4.2.2 Procedure-specific multimodal analgesia and enhanced recovery

Clinical comparisons are most developed in arthroplasty, but the results remain comparator- and pathway-sensitive. Adductor-canal and periarticular trials report pain or opioid advantages in some settings (24, 40, 41), whereas meta-analysis and noninferiority work does not consistently reproduce these differences when the control pathway is well optimized (32, 33). PENG and parasacral ischial plane trials extend the comparison to other hip and knee pathways (21, 46). In abdominal surgery, pooled evidence and a colorectal TAP-block trial have not established consistent superiority over standard bupivacaine (6, 47, 48). Shoulder studies likewise vary with the comparator, including heterogeneous syntheses (7, 23), noninferiority or early pain benefit (49, 50), and catheter comparisons that incorporate patient experience and resource use (51, 52). Orthopedic perineural evidence remains equivocal, although selected spine operations may show pain or opioid reductions (8), and lumbar fusion erector spinae plane studies extend this question (13). Breast-reconstruction studies also remain heterogeneous, so donor-site pain and early mobilization should be evaluated alongside opioid use. Future trials should prespecify a procedure-specific primary endpoint, standardize background analgesia and rescue thresholds, and stratify by baseline opioid exposure, surgical invasiveness, and discharge destination. Movement-evoked pain, opioid-related adverse effects, function, length of stay, readmission, and cost should be reported together; opioid reduction alone may not establish recovery benefit (22).

#### 4.2.3 Regional anesthesia and fascial-plane/peripheral nerve blocks

Block-level evidence is not interchangeable across anatomy. Volunteer and sciatic-block studies provide a framework for measuring pharmacodynamic duration (53). In shoulder surgery, results vary with plain bupivacaine, perineural dexamethasone, and continuous catheters (50, 51), with catheter comparisons also capturing patient experience and resource use (51). Recent upper-extremity trials reinforce this comparator dependence (54, 55). For fascial-plane blocks, volume, spread, fascial continuity, and access to the intended nerves shape analgesia. Abdominal-plane meta-analysis has not shown a reliable class-wide advantage (47), whereas selected spinal procedures may benefit from erector spinae approaches (56). Thoracic studies report reductions in some randomized settings (20, 57), but cardiac and enhanced-recovery studies remain procedure-dependent (58, 59). Paravertebral applications in liver resection and pediatric pectus repair broaden the settings (19, 60), while pooled thoracic evidence remains methodologically heterogeneous (61). Future trials should report ultrasound-confirmed technique, dose and volume, sensory mapping, movement-evoked and rebound pain, motor impairment, and direct comparisons with catheters or inexpensive adjuvants (62).

### 4.3 Limitations

As the first systematic bibliometric analysis on liposomal bupivacaine, this study objectively delineates the developmental trajectory from bench to bedside through quantitative and co-citation network analyses, offering valuable insights and future directions for both researchers and clinicians. Nonetheless, several potential limitations should be acknowledged. First, the data were retrieved exclusively from the Web of Science and Scopus databases, which may omit relevant studies indexed elsewhere and limit the overall comprehensiveness of the dataset. Second, the analytical workflows and tools were predominantly structured around English-language publications; overlooking non-English literature may impede a complete representation of global research activities and introduce potential language bias. Third, this bibliometric analysis did not perform granular risk-of-bias assessments on individual included studies, nor did it evaluate the methodological quality or strength of clinical evidence. Finally, citation accumulation inherently involves a time-lag effect, which may result in a delayed response when capturing the most nascent research frontiers or rapidly emerging clinical topics compared to real-time investigative developments.

## 5 Conclusion

In this comprehensive bibliometric analysis, we systematically evaluated an extensive body of literature using CiteSpace, VOSviewer, and the Bibliometrix R package. Our findings delineate three interrelated thematic domains: extended-release drug delivery systems, procedure-specific multimodal analgesia within Enhanced Recovery After Surgery (ERAS) pathways, and the utility of liposomal bupivacaine in peripheral nerve and fascial plane blocks. Arthroplasty continues to represent the most established clinical scenario, whereas shoulder surgery, breast reconstruction, spine surgery, and thoracic surgery reflect active, emerging frontiers of clinical expansion. Furthermore, the evidence base has progressively transitioned from early exploratory investigations to randomized controlled trials (RCTs), systematic reviews, and granular evaluations of clinical endpoints, including opioid consumption, functional recovery, length of stay, and safety profiles.

Nevertheless, bibliometric data reveal that although liposomal bupivacaine has garnered sustained and escalating momentum—particularly in postoperative analgesia—surges in publication volume and burst strength merely reflect academic attention and research activity rather than proven clinical superiority. Real-world efficacy remains highly heterogeneous, varying across surgical settings, anatomical injection sites, dosages, active comparators, and analgesic regimens, necessitating further high-quality RCTs for robust validation. Future investigations should prioritize multicenter, procedure-specific RCTs employing standardized administration protocols and focusing on patient-centered recovery outcomes, thereby elucidating precisely which clinical pathways derive tangible clinical benefits.

## Data Availability

The datasets presented in this study can be found in online repositories. The names of the repository/repositories and accession number(s) can be found in the article/Supplementary Material.
