# Supplementary material for: Research trends and hotspots of liposomal bupivacaine: a bibliometric analysis from 2000 to 2025
Source: Front Med (Lausanne). 2026 Oct 13;13:1955952. doi: 10.3389/fmed.2026.1955952 (PMC13710353; doi:10.3389/fmed.2026.1955952)
Supplement: Supplementary file 1 [file Table1.docx]

Table S1 Top 10 Most Productive Countries in Terms of Publications

| Country | Documents | Total Citations | Average Citations | TLS |
| --- | --- | --- | --- | --- |
| United States | 2075 | 63218 | 30.47 | 418 |
| China | 565 | 9186 | 16.26 | 107 |
| Canada | 178 | 17036 | 95.71 | 180 |
| United Kingdom | 127 | 9494 | 74.76 | 170 |
| India | 99 | 4929 | 49.79 | 52 |
| Italy | 97 | 2708 | 27.92 | 102 |
| Brazil | 82 | 2171 | 26.48 | 44 |
| South Korea | 79 | 1542 | 19.52 | 24 |
| Germany | 69 | 4141 | 60.01 | 105 |
| Spain | 56 | 2579 | 46.05 | 103 |

Table S2 Top 10 Most Productive Affiliations in Terms of Publications

| Organization | Documents | Total Citations | Average Citations | TLS |
| --- | --- | --- | --- | --- |
| Mayo Clinic | 105 | 2096 | 19.96 | 15 |
| Cleveland Clinic | 59 | 1743 | 29.54 | 19 |
| Duke Univ | 56 | 2246 | 40.11 | 21 |
| Sichuan Univ | 42 | 513 | 12.21 | 0 |
| Brigham and Women's Hospital | 37 | 2949 | 79.70 | 6 |
| University of California | 37 | 5730 | 154.86 | 20 |
| University of Toronto | 36 | 3198 | 88.83 | 29 |
| Harvard Med Sch | 30 | 1237 | 41.23 | 11 |
| Massachusetts General Hospital | 29 | 3951 | 136.24 | 16 |
| Stanford University | 28 | 1293 | 46.18 | 21 |

Table S3 Top 10 Most Productive Authors

| Author | Documents1 | Total Citations | Average Citations | TLS |
| --- | --- | --- | --- | --- |
| De Paula, Eneida | 40 | 1092 | 27.30 | 57 |
| Franz-montan, Michelle | 20 | 489 | 24.45 | 42 |
| Kohane, Daniel S. | 20 | 1162 | 58.10 | 0 |
| Turan, Alparslan | 17 | 302 | 17.76 | 0 |
| Joshi, Girish P | 16 | 471 | 29.44 | 6 |
| Onel, Erol | 16 | 1751 | 109.44 | 1 |
| Schwarzkopf, Ran | 16 | 259 | 16.19 | 1 |
| Zhang, Wensheng | 13 | 168 | 12.92 | 11 |
| Janis, Jeffrey E | 12 | 283 | 23.58 | 6 |
| Urman, Richard D | 12 | 1579 | 131.58 | 9 |

Table S4 Top 20 Keywords of the Top 10 Most Productive Countries

| Country | TOP Keuwords |
| --- | --- |
| United States | liposomal bupivacaine(270); pain management(156); pain(152); postoperative pain(136); bupivacaine(131); analgesia(125); Regional anesthesia(118); opioids(106); total knee arthroplasty(100); Opioid(96); enhanced recovery after surgery(88); multimodal analgesia(84); nerve block(62); ERAS(58); exparel(57); Anesthesia(56); pain control(53); Length of stay(46); acute pain(40); local anesthetic(37) |
| China | meta-analysis(63); liposomal bupivacaine(61); postoperative pain(52); total knee arthroplasty(51); ropivacaine(44); analgesia(41); postoperative analgesia(32); bupivacaine(29); pain(29); dexmedetomidine(26); pain management(26); nerve block(25); intercostal nerve block(18); pain control(18); Erector spinae plane block(16); Video-assisted thoracoscopic surgery(16); local anesthetics(15); transversus abdominis plane block(15); Anesthesia(14); dexamethasone(14) |
| Canada | analgesia(18); pain(15); nerve block(12); pain management(12); Regional anesthesia(11); Enhanced recovery(10); enhanced recovery after surgery(9); systematic review(9); ERAS(8); postoperative pain(8); multimodal analgesia(7); liposomal bupivacaine(6); opioids(6); Perioperative care(6); peripheral nerve block(6); surgery(6); Thoracic surgery(6); Adductor canal block(5); Anesthesia(5); postoperative(5) |
| United Kingdom | analgesia(14); enhanced recovery after surgery(13); pain management(8); pain(7); postoperative pain(7); liposomal bupivacaine(6); Perioperative care(6); Opioid(5); Regional anesthesia(5); acute pain(4); ERAS(4); knee(4); liposomes(4); nerve block(4); opioids(4); Shoulder arthroplasty(4); total knee arthroplasty(4); arthroplasty(3); chronic pain(3); Enhanced recovery(3) |
| India | liposomes(11); analgesia(8); drug delivery(8); pain(8); Nanocarriers(7); bupivacaine(6); nerve block(6); enhanced recovery after surgery(5); multimodal analgesia(5); postoperative pain(5); ERAS(4); liposome(4); Nanomedicine(4); nanotechnology(4); pain management(4); cancer(3); Cardiac surgery(3); DepoFoam(3); knee(3); liposomal bupivacaine(3) |
| Italy | pain(12); Anesthesia(10); postoperative pain(9); pain management(8); analgesia(7); Regional anesthesia(5); TAP block(5); total knee arthroplasty(5); ERAS(4); Guidelines(4); liposomes(4); microparticle(4); nerve block(4); transversus abdominis plane block(4); Bariatric surgery(3); complications(3); enhanced recovery after surgery(3); epidural(3); femoral nerve block(3); knee(3) |
| Brazil | liposomes(17); drug delivery(14); local anesthetics(13); liposome(8); bupivacaine(6); dentistry(6); local anesthetic(6); Lidocaine(4); ropivacaine(4); analgesia(3); Benzocaine(3); butamben(3); Cyclodextrin(3); cyclodextrins(3); cytokine(3); Cytotoxicity(3); Ionic gradient liposomes(3); liposomal bupivacaine(3); local anesthesia(3); mepivacaine(3) |
| South Korea | postoperative pain(14); pain management(8); nerve block(6); local anesthetics(5); pain(4); postoperative analgesia(4); total knee arthroplasty(4); acute pain(3); bupivacaine(3); Colorectal cancer(3); enhanced recovery after surgery(3); femoral nerve block(3); hydrogel(3); knee(3); Laparoscopy(3); Lidocaine(3); nanoparticle(3); periarticular injection(3); Regional anesthesia(3); ropivacaine(3)Colorectal surgery(2); drug delivery system(2); Erector spinae plane block(2); Interscalene brachial plexus block(2) |
| Germany | postoperative pain(6); pain management(5); analgesia(4); pain(4); bupivacaine(3); Enhanced recovery(3); liposomes(3); phospholipids(3); acute postoperative pain(2); Anesthesia(2); Cardiac surgery(2); chronic pain(2); Guidelines(2); Laparoscopy(2); liposomal bupivacaine(2); local anesthesia(2); Lumbar(2); meta-analysis(2); Microfluidics(2); Nanomedicine(2) |
| Spain | postoperative pain(11); bupivacaine(7); pain(4); drug delivery(3); enhanced recovery after surgery(3); Guidelines(3); local anesthetics(3); meta-analysis(3); pain management(3); Epidural analgesia(2); intercostal nerve block(2); intrathecal morphine(2); knee(2); Laparoscopic bariatric surgery(2); liposome(2); local infiltration analgesia(2); multimodal analgesia(2); opioids(2); prilocaine(2); Regional anesthesia(2) |

Table S5 Top 20 Keywords of the Top 10 Most Productive Affiliations

| Country | TOP Keuwords |
| --- | --- |
| Mayo Clinic | liposomal bupivacaine(10); pain(6); bupivacaine(4); enhanced recovery after surgery(4); opioids(4); exparel(3); pain management(3); total hip arthroplasty(3); complications(2); ERAS(2); Fusion(2); interscalene nerve block(2); Liposomal(2); mastectomy(2); multimodal analgesia and anesthesia(2); Opioid(2); periarticular injection(2); Perioperative care(2); postoperative pain(2); Regional anesthesia(2) |
| Cleveland Clinic | liposomal bupivacaine(11); bupivacaine(5); pain management(4); postoperative pain(4); analgesia(3); Anesthesia(3); multimodal analgesia(3); pain(3); total knee arthroplasty(3); Epidural analgesia(2); exparel(2); Length of stay(2); liposomes(2); local anesthetics(2); nerve block(2); Opioid consumption(2); opioids(2); Pediatrics(2); Postsurgical pain(2); surgery(2) |
| Duke Univ | liposomal bupivacaine(10); bupivacaine(7); postoperative pain(5); liposomes(4); pain(4); analgesia(3); Anesthesia(3); ankle arthritis(2); ankle arthroplasty(2); local anesthetics(2); multimodal analgesia(2); nerve block(2); nerve blocks(2); nonopioid analgesic(2); outcomes studies(2); pain control(2); Regional anesthesia(2); total knee arthroplasty(2); Adductor canal(1); admission status(1) |
| Sichuan Univ | liposomal bupivacaine(4); total knee arthroplasty(4); meta-analysis(3); pain control(3); adjuvant(2); analgesia(2); local anesthesia(2); QX-OH(2); wound infiltration(2); Adult anaesthesia(1); adverse reactions(1); anaesthetics(1); bupivacaine(1); carrier-free formulation(1); cocktail analgesia(1); dexamethasone(1); drug delivery system(1); drug microcrystals(1); Extended-release(1); FAERS database(1) |
| Brigham and Women's Hospital | pain management(6); analgesia(4); multimodal analgesia(4); opioids(4); enhanced recovery after surgery(3); pain(3); Anesthesia(2); Enhanced recovery(2); ERAS(2); Evidence-based medicine(2); intraoperative care(2); liposomal bupivacaine(2); local infiltration analgesia(2); Opioid-free anesthesia(2); postoperative pain(2); preoperative care(2); Regional anesthesia(2); surgery(2); systematic review(2); 1 Layer(1) |
| University of California | analgesia(3); Enhanced recovery(3); Opioid(3); pain(3); acute pain(2); bupivacaine(2); Colectomy(2); Education(2); Ileus(2); multimodal analgesia(2); pain management(2); postoperative analgesia(2); Proctectomy(2); acute pain service(1); Anesthesia(1); anesthesiology(1); animal research(1); animal welfare(1); breast cancer(1); burn(1) |
| University of Toronto | analgesia(6); nerve block(4); Regional anesthesia(4); local anesthetic(3); pain(3); pain management(3); postoperative pain(3); acute pain(2); enhanced recovery after surgery(2); multimodal analgesia(2); peripheral nerve block(2); postoperative(2); Rebound pain(2); regional anaesthesia(2); shoulder(2); Adductor canal block(1); adverse events(1); ambulatory surgical procedures(1); Anaesthesia(1); analgesics(1) |
| Harvard Med Sch | liposome(5); liposomal bupivacaine(4); local anesthesia(4); Tetrodotoxin(4); analgesia(3); bupivacaine(3); nerve block(3); acute pain(2); enhanced recovery after surgery(2); exparel(2); local anesthetics(2); Anaesthesia(1); Analgesia obstetrical(1); Analgesics opioid(1); Anesthesia(1); Anesthesia obstetrical(1); arthroplasty(1); biocompatibility(1); Cesarean section(1); Colectomy(1) |
| Massachusetts General Hospital | postoperative pain(4); acute pain(3); Enhanced recovery(3); analgesia(2); Colectomy(2); drug delivery(2); Ileus(2); Opioid(2); opioids(2); Proctectomy(2); Regional anesthesia(2); Thoracic surgery(2); adult cardiac surgery(1); analgesics(1); Anesthesia(1); Anesthetic choice(1); animal model(1); Anterior cervical discectomy and fusion(1); biocompatibility(1); Biotechnology(1) |
| Stanford University | pain management(3); multimodal analgesia(2); Prehabilitation(2); transversus abdominis plane block(2); analgesia(1); analgesics(1); Anesthesia(1); animal research(1); animal welfare(1); buprenorphine(1); Cesarean delivery(1); Composite score rating for pulmonary resection(1); double-lumen tube(1); Enhanced recovery after surgery (ERAS)(1); Enhanced recovery after thoracic surgery(1); enhanced recovery in thoracic surgery(1); Exercise(1); Expectations(1); extracorporeal circulation(1); extracorporeal membrane oxygenation(1) |

Table S6 Top20 Keywords of the Top 10 Most Productive Authors

| Country | TOP Keuwords |
| --- | --- |
| De Paula, Eneida | liposomes(12); local anesthetics(11); drug delivery(9); liposome(6); dentistry(4); local anesthetic(4); ropivacaine(4); Benzocaine(3); bupivacaine(3); butamben(3); Cyclodextrin(3); cyclodextrins(3); local anesthesia(3); mepivacaine(3); cytokine(2); Cytotoxicity(2); drug-delivery(2); Etidocaine(2); Ionic gradient liposomes(2); Lidocaine(2) |
| Franz-montan, Michelle | liposomes(6); local anesthetics(5); dentistry(4); drug delivery(4); liposome(4); cyclodextrins(3); permeation(3); Topical anesthesia(3); anesthetic efficacy(2); Benzocaine(2); butamben(2); local anesthesia(2); local anesthetic(2); mepivacaine(2); polymer(2); ropivacaine(2); sustained release(2); Anesthesia(1); Articaine(1); bioassay approaches(1) |
| Kohane, Daniel S. | liposome(6); Tetrodotoxin(5); bupivacaine(3); local anesthesia(3); drug delivery(2); gold nanorod(2); local anesthetics(2); Myotoxicity(2); Neurotoxicity(2); Phototriggering(2); biocompatibility(1); Concanavalin A(1); controlled drug release(1); Crosslinking(1); DepoFoam(1); dexmedetomidine(1); Drug nanoparticle(1); drug-aptamer complex(1); Exparel (TM)(1); hyaluronic acid(1) |
| Turan, Alparslan | postoperative pain(3); analgesia(2); Anesthesia(2); bupivacaine(2); Epidural analgesia(2); liposomal bupivacaine(2); liposomes(2); local anesthetics(2); multimodal analgesia(2); pain(2); Regional anesthesia(2); Abdominal surgery(1); abdominal wall blocks(1); airway blocks(1); Anesthetics(1); blocks(1); cardiac anaesthesia(1); Cardiac surgery(1); chest wall blocks(1); conduction(1) |
| Joshi, Girish P | analgesia(2); multimodal analgesia(2); Cesarean delivery(1); Complementary Therapies(1); complications(1); Emergency surgery(1); Epidemiology(1); Evidence-based medicine(1); femoral nerve block(1); laparoscopic hysterectomy(1); liposomal bupivacaine(1); obstetrics(1); open total abdominal hysterectomy(1); Opioid-sparing(1); pain(1); pain management(1); Periarticular infiltration(1); postoperative pain(1); postoperative pain management(1); Readmission(1) |
| Onel, Erol | DepoFoam bupivacaine(4); wound infiltration(4); analgesic(3); Clinical trial(3); analgesia(2); Anesthesia(2); bunionectomy(2); bupivacaine(2); Infiltration(2); liposome bupivacaine(2); Mammaplasty(2); pain(2); Postsurgical analgesia(2); Postsurgical pain(2); total knee arthroplasty(2); 1(1); breast augmentation(1); bupivacaine HCl(1); DepoFoam(1); exparel(1) |
| Schwarzkopf, Ran | total hip arthroplasty(6); total knee arthroplasty(6); pain(4); Opioid consumption(3); Adductor canal block(2); liposomal bupivacaine(2); opioids(2); pain management(2); patient-reported outcomes(2); quality outcomes(2); Tourniquet(2); value-based care(2); chronic opioid(1); chronic opioid use(1); complications(1); dexamethasone(1); dual mobility(1); Fall(1); femoral head size(1); functional status(1) |
| Zhang, Wensheng | long-acting analgesia(3); postoperative analgesia(3); QX-OH(3); Levobupivacaine(2); local toxicity(2); Long-acting local anesthesia(2); Long-acting local anesthetic(2); slow release(2); carrier-free formulation(1); drug microcrystals(1); fixed-dose combination (FDC)(1); Injectable thermo-sensitive hydrogel(1); investigational new drug(1); liposomal bupivacaine(1); liposome bupivacaine(1); Liquid chromatography-mass spectrometry(1); local anesthetic(1); local anesthetic toxicity(1); local anesthetics(1); Nanocrystal(1) |
| Janis, Jeffrey E | Abdominal core health(1); abdominal wall reconstruction(1); Holistic care(1); Multidisciplinary care(1); multimodal analgesia(1); pain management(1); plastic surgery(1) |
| Urman, Richard D | analgesia(2); liposomal bupivacaine(2); multimodal analgesia(2); pain management(2); Regional anesthesia(2); Adjuvant analgesics(1); adjuvants(1); adult anesthetics(1); Ambulatory(1); Anesthesia(1); breast augmentation(1); breast surgery(1); bupivacaine(1); chronic pain(1); Cosmetic surgery(1); Delivery systems(1); enhanced recovery after surgery(1); ERAS(1); exparel(1); ketamine(1) |

Data_merge code：

The core code used in this study has been made open source and is publicly available at the following repository:

[https://github.com/Enjoyability/Data_merge.git](https://github.com/Enjoyability/Data_merge.git?utm_source=gemini).
